# Molecular characterization, tissue tropism, and genetic variability of the novel *Mupapillomavirus* type HPV204 and phylogenetically related types HPV1 and HPV63

**DOI:** 10.1371/journal.pone.0175892

**Published:** 2017-04-20

**Authors:** Anja Šterbenc, Lea Hošnjak, Diego Chouhy, Elisa M. Bolatti, Anja Oštrbenk, Katja Seme, Boštjan J. Kocjan, Boštjan Luzar, Adriana A. Giri, Mario Poljak

**Affiliations:** 1 Institute of Microbiology and Immunology, Faculty of Medicine, University of Ljubljana, Ljubljana, Slovenia; 2 Virology Area, School of Biochemistry and Pharmaceutical Sciences, Rosario National University, Rosario, Argentina; 3 Institute of Pathology, Faculty of Medicine, University of Ljubljana, Ljubljana, Slovenia; Albert Einstein College of Medicine, UNITED STATES

## Abstract

HPV204 is the only newly identified *Mupapillomavirus* (*Mu*-PV) type in more than a decade. To comprehensively characterize HPV204, we performed a detailed molecular analysis of the viral genome and evaluated its clinical relevance in comparison to the other *Mu*-PVs, HPV1 and HPV63. The 7,227-bp long genome of HPV204 exhibits typical genomic organization of *Mu*-PVs with eight open reading frames (ORFs) (E6, E7, E1, E2, E8, E4, L2, and L1). We developed three type-specific quantitative real-time PCRs and used them to test a representative collection (*n* = 1,006) of various HPV-associated benign and malignant neoplasms, as well as samples of clinically normal cutaneous, mucosal, and mucocutaneous origins. HPV204, HPV1, and HPV63 were detected in 1.1%, 2.7%, and 1.9% of samples tested, respectively, and were present in skin and mucosa, suggesting dual tissue tropism of all *Mu*-PVs. To evaluate the etiological role of *Mu*-PVs in the development of HPV-associated neoplasms, *Mu*-PV viral loads per single cell were estimated. HPV1 and HPV63 were present in high viral copy numbers in 3/43 and 1/43 cutaneous warts, respectively, and were identified as the most likely causative agents of these warts. HPV204 viral load was extremely low in a single HPV204-positive cutaneous wart (7.4 × 10^−7^ viral copies/cell). Hence, etiological association between HPV204 and the development of cutaneous warts could not be established. To the best of our knowledge, this is the first study to evaluate the genetic variability of *Mu*-PVs by sequencing complete LCR genomic regions of HPV204, HPV1, and HPV63. We detected several nucleotide substitutions and deletions within the LCR genomic regions of *Mu*-PVs and identified two genetic variants of HPV204 and HPV63 and five genetic variants of HPV1.

## Introduction

Human papillomaviruses (HPVs) are small, double-stranded and highly diverse DNA viruses of the *Papillomaviridae* family, which typically infect human epithelial cells [1]. HPVs are etiologically associated with the development of various benign and malignant neoplasms in humans; however, the majority of HPV infections are transient and/or latent [2, 3]. Based on the identity of the complete L1 gene nucleotide sequences, HPVs are hierarchically classified into genera, species, and types. HPV types exhibiting at least 60% nucleotide sequence identity within the L1 gene belong to the same genus, whereas HPV types with 60 to 70% nucleotide sequence similarity constitute different viral species [1]. As of March 31st, 2017, 206 HPV types were officially recognized by the HPV Reference center at the Karolinska Institute in Sweden (http://www.hpvcenter.se/html/refclones.html). Currently, all HPV types are classified into five genera: *Alphapapillomavirus* (*Alpha*-PV), *Betapapillomavirus* (*Beta*-PV), *Gammapapillomavirus* (*Gamma*-PV), *Mupapillomavirus* (*Mu*-PV), and *Nupapillomavirus* (*Nu*-PV) [4, 5].

Contrary to the rapidly growing *Beta-* and *Gamma*-PV genera, the *Mu*-PV genus currently consists of only three HPV types: HPV1, HPV63, and HPV204 [5, 6]. In 1980, HPV1 was isolated and cloned from tissue specimens of deep plantar warts [7, 8] and its complete nucleotide sequence was obtained 2 years later (GeneBank accession numbers V01116 and NC_001356) [9]. HPV63 (GeneBank accession number X70828) was originally identified in punctate keratotic lesions of the foot in 1993 [10]. At the nucleotide level, HPV63 displayed 69.0% sequence homology with the HPV1 L1 open reading frame (ORF) and thus constituted a distinct species (*Mu*-2) within the *Mu*-PV genus [10]. Identification and preliminary characterization of a novel *Mu*-PV type HPV204 has been recently described by our research group [6, 11]. Briefly, in 2009 we identified a partial 200-bp L1 gene sequence exhibiting the highest sequence similarity with *Mu*-PVs HPV1 and HPV63 in a skin swab sample using the broad-spectrum FAP primer system [11–13]. In 2014, the complete viral genome of a putatively novel *Mu*-PV type was amplified from a swab of the anal canal obtained from a 36-year-old male patient with HPV53-positive anal warts. The corresponding amplicon was cloned into a plasmid vector and sequenced by primer walking strategy [6]. Nucleotide sequence (GeneBank accession number KP769769) of the complete viral genome was submitted to the HPV Reference Center at the Karolinska Institute. The sequence was reconfirmed and the novel *Mu*-PV type was officially assigned the number HPV204 in January 2015 (http://www.hpvcenter.se/html/refclones.html). According to the International Committee on Taxonomy of Viruses (ICTV), HPV204 constitutes a distinct species (*Mu*-3) within the *Mu*-PV genus, since the L1 nucleotide sequence of HPV204 exhibits only 66.3% and 66.7% sequence identity with L1 genes of HPV1 and HPV63, respectively (https://talk.ictvonline.org/files/ictv_official_taxonomy_updates_since_the_8th_report/m/animal-dna-viruses-and-retroviruses?pi4565=2). HPV204 represents the only novel *Mu*-PV type in more than a decade, despite the use of several modern molecular methods to identify potentially novel HPVs in recent years [6, 11, 14].

To date, tissue tropism of HPV204 has not been described in peer-reviewed literature, whereas it has been shown that HPV1 and HPV63 typically infect keratinocytes of eccrine ducts in the palmoplantar skin and are hence considered cutaneotrophic [15, 16]. HPV1 was also identified in an oral sample in a recent metagenomics study [17], suggesting that *Mu*-PVs exhibit broader tissue tropism than previously expected.

Amplification and sequencing of eight HPV1 L1 amplicons obtained from scrapings of psoriatic lesions revealed a single nucleotide polymorphism (SNP) (A5793C) within the L1 gene of HPV1 [18]. However, the long control region (LCR) exhibits the highest level of diversity among HPVs [19] and its variability in *Mu*-PVs has not been described prior to this study.

In this study, we present a detailed molecular characterization of HPV204 in comparison to HPV1 and HPV63. To determine the tissue tropism and potential clinical significance of *Mu*-PVs, we systematically investigated the prevalence of HPV204, HPV1, and HPV63 and determined corresponding viral loads in *Mu*-PV–positive samples in a representative collection of clinical specimens commonly associated with HPV infection, using three newly developed quantitative type-specific real-time PCRs (RT-PCRs). In addition, to the best of our knowledge, this is the first study to assess genetic diversity of *Mu*-PVs by amplifying and sequencing their complete LCR genomic regions.

## Materials and methods

### Molecular characterization of HPV204, HPV1, and HPV63

We used the ORF Finder Tool (https://www.ncbi.nlm.nih.gov/gorf/gorf.html) to identify distinct ORFs of HPV204. In addition, viral genomic regions and protein functional domains were characterized in detail using previously published data and several freely available online applications [20–25]. The same protocol was used to characterize as-yet undescribed functional domains of HPV1 and HPV63 [9, 10, 26, 27].

### HPV204, HPV1, and HPV63 type-specific quantitative RT-PCRs

All type-specific primer pairs and probes (Table 1) were designed using the freely available online application Primer3Plus (http://primer3plus.com/cgi-bin/dev/primer3plus.cgi). To avoid potential cross-reactivity with non-targeted sequences, the specificity of each primer and probe was verified using NCBI Blast (https://blast.ncbi.nlm.nih.gov/Blast.cgi).

**Table 1:** General characteristics of primers and probes designed for HPV204, HPV1, and HPV63 type-specific quantitative real-time PCRs.

HPV (targeted gene)	Primer/probe^a^ nucleotide sequence (5′–3′)	Amplicon size (bp)	Nucleotide position
**HPV204 (L1)**	TTTAATCCTGAAACAGAACGCTT	133	5,461–5,483
	ATCTAGCAGGATTTTCCACATCA		5,593–5,571
	FAM-AAGTAGTGTTACCGGGCATCCTA-BHQ		5,532–5,554
**HPV1 (L1)**	AGCAACATGCAAATATCCTGATT	148	6,103–6,125
	TTGTGGGACTGCCTCCTTATC		6,250–6,230
	YAK-GCGAGCAAATGTATACCAGGCACT-BHQ		6,180–6,203
**HPV63 (E2)**	TCCTGTCAATAGCTCCCCACT	108	3,219–3,239
	GACCCCTTCGTCTCTGCTTT		3,326–3,307
	FAM-ACACCAACCCAGCCACCCAAG-BHQ		3,263–3,283

Genbank accession numbers: KP769769 (HPV204), V01116 (revised; HPV1), and X70828 (HPV63).

^a^ Real-time amplification of targeted *Mu*-PVs was monitored using 5′ dually labeled hydrolysis probes.

The HPV204 type-specific quantitative RT-PCR was performed using a QuantiTect Probe PCR kit (Qiagen, Hilden, Germany). The reaction mixture consisted of 5 μl of sample DNA, 10 μl of 2X QuantiTect Probe RT-PCR Master mix, 0.4 μM of each primer, 0.2 μM of the probe, and water up to the final reaction volume of 20 μl. The assay was performed on a LightCycler 480 II RT-PCR Instrument (Roche Diagnostics, Mannheim, Germany) under the following cycling conditions: an initial 15 min incubation step at 95°C (ramp rate 4.4°C/s), followed by 45 cycles of (i) 15 s at 95°C (2.2°C/s), (ii) 60 s at 60°C (2.2°C/s), and a final cooling step with a 30 s hold at 40°C (2.2°C/s). The fluorescence signal was measured during each annealing/extension step on the 510 nm channel.

HPV1 and HPV63 type-specific quantitative RT-PCRs were performed using a LightCycler480 Probes kit (Roche Diagnostics). The reaction mixture for the detection of HPV1 consisted of 5 μl of sample DNA, 10 μl of 2X LightCycler480 Probes Master, 0.6 μM of each primer, 0.4 μM of the probe, and water up to the final reaction volume of 20 μl. The reaction mixture for the detection of HPV63 consisted of 5 μl of sample DNA, 10 μl of 2X LightCycler480 Probes Master, 0.5 μM of each primer, 0.2 μM of the probe, and water up to the final reaction volume of 20 μl. Both assays were performed on a LightCycler 480 II RT-PCR Instrument (Roche Diagnostics), under the following cycling conditions: an initial 10 min incubation step at 95°C (4.4°C/s), followed by 45 cycles of (i) 10 s at 95°C (4.4°C/s), (ii) 30 s at 60°C (2.2°C/s), (iii) 1 s at 72°C (4.4°C/s), and a final cooling step with a 30 s hold at 40°C (2.2°C/s). Fluorescence signals of HPV1 and HPV63 were measured during each extension step on 560 and 510 nm channels, respectively.

Triplicates of 10-fold serial dilutions of HPV204, HPV1, and HPV63 reference plasmids, ranging from 1 × 10^9^ to 1 × 10^−1^ DNA copies/reaction, were used to evaluate analytical sensitivity of each type-specific RT-PCR. Dynamic ranges of HPV204 and HPV63 RT-PCRs were nine orders of magnitude, and enabled reliable discrimination of 10^9^ to one viral copy/reaction, whereas the dynamic range of the HPV1 RT-PCR was eight orders of magnitude and reliably discriminated 10^9^ to 10 viral copies/reaction. The detection limit of HPV204 and HPV63 RT-PCRs was at least one viral copy/reaction and for HPV1 RT-PCR at least 10 viral copies/reaction. The standard curve correlation coefficients (*R*^2^) calculated were as follows: 0.998 for HPV204 RT-PCR and 0.999 for both HPV1 and HPV63 RT-PCRs. All RT-PCRs had high amplification efficiencies of 100% (HPV204), 94.4% (HPV1), and 99.2% (HPV63). Specificity and cross-reactivity of HPV204, HPV1, and HPV63 RT-PCRs were evaluated using serial dilutions of *Mu*-PV reference plasmids, ranging from 10^9^ to 10^4^ DNA copies/reaction: HPV204 RT-PCR was evaluated using HPV1 and HPV63 reference plasmids, HPV1 RT-PCR using HPV204 and HPV63 reference plasmids, and HPV63 RT-PCR using HPV204 and HPV1 reference plasmids. In addition, the specificity of all HPV204, HPV1, and HPV63 RT-PCR amplicons was confirmed by direct Sanger sequencing.

### Tissue tropism and viral load of *Mu*-PVs

Using three type-specific quantitative RT-PCRs, the clinical significance and tissue tropism of *Mu*-PVs was assessed by testing a representative collection of clinical specimens most commonly associated with HPV infection (*n* = 1,006). The sample collection included histologically confirmed tissue specimens of benign neoplasms of cutaneous (cutaneous warts), mucocutaneous (anogenital warts) and mucosal origin (laryngeal and conjunctival papillomas) as well as mucosal (cervical, oral/oropharyngeal, and conjunctival squamous cell carcinoma (SCC)) and cutaneous (SCC and basal cell carcinoma (BCC) of the skin) HPV-related malignant neoplasms. To determine whether *Mu*-PVs are also present in clinically normal cutaneous, mucocutaneous, and mucosal epithelia, eyebrow hair follicles, swabs of the penile surface, swabs of the anal canal (obtained from patients with hemorrhoids, anal fissure, and anal warts), swabs of the oral cavity, nasopharyngeal swabs, and cervical swabs were also included in the sample collection. To evaluate the potential role of *Mu*-PVs in the development of different neoplasms, HPV204, HPV1, and/or HPV63 viral loads were estimated in all HPV204-, HPV1-, and HPV63-positive tissue samples. The concentration of beta-globin was determined using a quantitative RT-PCR amplifying a 150-bp fragment of the human beta-globin gene, and was used in combination with HPV type-specific RT-PCRs to determine the ratio between the number of viral copies and human diploid cells, as described previously [24, 28].

The presence of other and/or additional mucosal and cutaneous wart-associated HPV types was determined using two previously described in-house PCR protocols [24, 25]. Briefly, we used primer pair HVP2/B5 to detect cutaneous *Alpha*- (HPV2, HPV3, HPV10, HPV27, HPV28, HPV29, HPV57, and HPV77), *Mu*- (HPV1 and HPV63), and *Nu*-PVs (HPV41), whereas a real-time PCR with a primer set HP1,3_F2/HP2_F2/HP_iLC and HP_LNA probe was used to detect low-risk *Alpha*-PVs HPV6 and HPV11. For both PCR protocols, 5 μl of sample DNA per 25 μl of reaction mixture was used. Amplicons obtained with a primer pair HVP2/B5 were analyzed on a 2.0% agarose gel. All specific amplicons were subsequently sequenced and HPV types were determined according to the NCBI Blast analysis (https://blast.ncbi.nlm.nih.gov/Blast.cgi).

### Amplification and sequencing of LCR genomic regions of HPV204, HPV1, and HPV63

In order to amplify complete LCR genomic regions of *Mu*-PVs, HPV204-, HPV1-, and HPV63-specific primer pairs (S1 Table) were designed using the Primer3Plus application. Single primer pairs were used for the amplification of the 510-bp and 555-bp long LCR genomic regions of HPV204 and HPV63, respectively. If no PCR specific amplicon was obtained, several overlapping primer pairs were additionally used in different combinations to obtain shorter PCR amplicons. Three sets of overlapping primers were initially used to amplify the complete LCR genomic region of HPV1 (979-bp) and up to seven additional primer pair combinations were used if the initial PCRs yielded no specific amplicons. Using FastStart High Fidelity PCR System (Roche Diagnostics), the reaction mixture consisted of 5 μl of sample DNA, 2.5 μl of 10 x FastStart High Fidelity Reaction Buffer, 200 μM of dNTP mix, 0.4 μM of each primer, 1.25 U of FastStart High Fidelity Enzyme Blend, and water up to the final reaction volume of 25 μl. Amplification of the LCR genomic region of all *Mu*-PVs was performed on a Veriti Thermal Cycler (Thermo Fisher Scientific, Waltham, MA, USA) under the following conditions: an initial incubation at 95°C for 2 min, followed by 45 cycles of (i) 30 s at 95°C, (ii) 30 s at 54°C, (iii) 30 s at 72°C, and 7 min incubation (final elongation) at 72°C with a final cooling step at 4°C. All PCR amplicons were evaluated on a 2.0% agarose gel and sequenced in both directions using the same primers as for PCR. Analytical sensitivity of HPV LCR type-specific PCRs was evaluated using 10-fold serial dilutions of HPV204, HPV1, and HPV63 reference plasmids, ranging from 1 × 10^9^ to 1 × 10^−1^ DNA copies/reaction. The detection limit of shorter PCR amplicons (209-bp to 359-bp) was determined to be 10 viral copies/reaction for HPV204 and HPV63, and 100 viral copies/reaction for HPV1.

Complete LCR genomic regions of HPV204, HPV1 and HPV63 were assembled using the Vector NTI Advance v11 program package (Invitrogen, Carlsbad, CA, USA). To identify potential SNPs and/or deletions, complete LCR genomic regions were subsequently aligned with HPV204, HPV1, and HPV63 reference sequences obtained from the Papillomavirus Episteme (PaVE) (https://pave.niaid.nih.gov/), and with all other available *Mu*-PV sequences in the Genbank database (Genbank accession numbers NC_001356 and U06714, both HPV1).

### Ethics statement

Our study was conducted in compliance with the Helsinki Declaration. A total of 1,006 clinical samples were collected either in our past or ongoing studies, which were all approved by the Ethics Committee of the Ministry of Health of Republic of Slovenia (consent numbers 34/11/06, 45/04/07, 131/06/07, 174/05/09, 83/11/09, 97/11/09, 100/12/09, 53/09/10, 109/08/12, and 63/10/13). None of the samples tested were obtained solely for the purpose of this study. All patients provided written informed consent prior to the beginning of the study. To assure patient confidentiality, all samples were coded and analyzed anonymously. The protocol and the use of all DNA samples were approved by the Institutional Review Board of the Institute of Microbiology and Immunology, Faculty of Medicine, University of Ljubljana.

## Results and discussion

### Molecular characterization of HPV204 in comparison to HPV1 and HPV63

**Fig 1:**
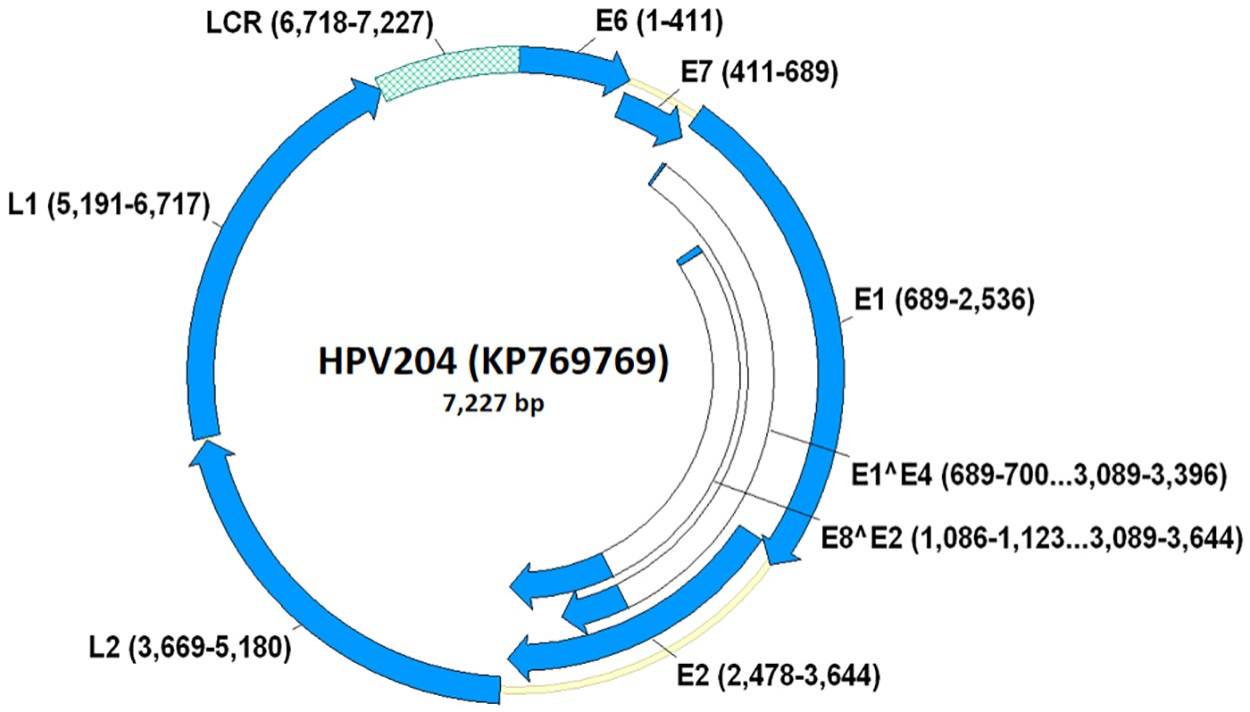
Genomic organization of HPV204 with genomic positions of viral early (E6, E7, E1, E2, E8, and E4) and late genes (L1 and L2). The non-coding long control region (LCR) is positioned between the L1 and E6 genes.

This study presents a detailed characterization of the complete genome of the novel *Mu*-PV HPV204, which is only the third officially recognized *Mu*-PV to date. As shown in Fig 1, HPV204 (7,227-bp) exhibits a genomic organization similar to those of previously described members of the *Mu*-PV genus (HPV1: 7,816-bp, HPV63: 7,348-bp) [6, 9, 10], with ORFs for six early (E6, E7, E1, E2, E8, and E4) and two late genes (L1 and L2), and a GC ratio of 37.8%. Genomic positions of viral genes and non-coding LCR genomic regions of *Mu*-PVs are presented in S2 Table.

The most important putative viral motifs and functional domains of *Mu*-PVs are presented in S3 Table. E6 proteins of all *Mu*-PVs contain two zinc-binding domains, which are crucial for the preservation of structural and biological functions of the E6 oncoprotein [29, 30]. Interestingly, the E7 zinc-binding domain of HPV204 (CXXC(X)_28_CXXC) is shorter by one amino acid (aa) when compared to the same motif in most HPVs. In addition to HPV204, this particular domain was only identified in HPV90 (*Alpha*-PV), whereas it is more commonly found in E7 proteins of animal PVs (data not shown). All *Mu*-PVs contain a pRB-binding site within their E7 proteins and are thus capable of inactivating the cell retinoblastoma protein (pRB) [31].

At the N-termini of the E1 protein, HPVs typically contain a highly conserved bipartite-like nuclear localization signal (NLS) and a leucine-rich Crm1-dependant nuclear export signal (NES), which together enable shuttling of the E1 protein between the cell nucleus and cytoplasm [32–34]. As shown in S3 Table, both motifs are present in E1 proteins of all *Mu*-PVs; however, we have identified an aa substitution (L94I) in the first position of NES of HPV1. The E1 proteins of all *Mu*-PVs contain several cdk-phosphorylation sites. In addition, a consensus cyclin-binding motif (RXL) was identified at the N-termini of the E1 proteins of HPV204 and HPV1, whereas HPV63 harbors a R117K substitution in the first position of its cyclin-binding motif. The cyclin-binding motif is necessary for interaction between the E1 and cyclin/cyclin-dependent kinase (cdk) complex and it has been shown that mutations in the consensus cyclin-binding motif and/or cdk-phosphorylation site(s) significantly reduce initiation of viral DNA replication [35–37]. In addition, the E1 proteins of all *Mu*-PVs contain a conserved ATP-binding site, which has two important structural roles: (i) it is crucial for formation of the E2-binding surface, and (ii) upon binding of ATP-Mg, it facilitates conformational changes in the E1 protein that occur during binding to the origin of replication [38].

As shown in S3 Table, we have identified a conserved leucine zipper domain, which is required for dimerization of the protein [39], at the C-terminus of the HPV204 E2 protein. Interestingly, although no conserved leucine zipper domain was observed at the C-terminus of the HPV1 E2 protein, one was identified at its N-terminus. According to the detailed analysis of all available HPV E2 reference aa sequences obtained from PaVE, HPV1 represents the only HPV type that harbors a leucine zipper domain at the N-terminus (data not shown). In contrast, no conserved leucine zipper domain was observed in the E2 protein of HPV63.

In addition to the full-length E2 protein, we identified a fusion protein E8^E2 consisting of the E8 gene product and C-terminal domain of the E2 gene. The E8^E2 protein not only inhibits viral transcription and replication of high-risk *Alpha*-PVs but it also suppresses genome replication of HPV1 and HPV8 (*Beta*-PV) in human keratinocytes via interaction with NCoR/SMRT corepressor complexes [40, 41]. As shown previously, the KLK motif of the E8 protein, which is required for the NCoR/SMRT-E8^E2 interaction, is highly conserved among all members of the *Mu*-PV genus [41, 42].

Similar to other HPVs, E4 ORFs of all members of the *Mu*-PV genus are located within the larger E2 ORFs and have their own start codons (Fig 1) [9, 10, 25, 43, 44]. However, identification of characteristic donor (AAG/GUASNR) and acceptor (GUYACYAG/YU) RNA splicing sites suggests that E4 proteins of all *Mu*-PVs are translated from a spliced E1^E4 mRNA with the initiation codon for E4 gene expression originating from the E1 ORF [24, 25, 43–45]. E1^E4 proteins of HPV204, HPV1, and HPV63 contain 11.5% (aa 15/131), 11.2% (aa 15/134), and 11.0% (aa 17/154) proline residues, respectively.

A consensus furin cleavage motif as well as a transmembrane-like domain [27] were identified at the N-termini of the L2 proteins of all *Mu*-PVs (S3 Table). Both domains are crucial for HPV infectivity because cleavage of the L2 protein at the highly conserved furin cleavage site promotes conformational changes of the viral capsid, while the transmembrane-like domain enables translocation of the viral genome across the endosomal membrane [27, 46]. In addition, a single canonical polyadenylation site necessary for regulation of early viral transcripts [47] is located at N-termini of L2 proteins of all *Mu*-PVs, whereas HPV1 contains an additional polyadenylation site in the middle of L2 ORF [9].

The LCR genomic region of HPV204 is positioned between the L1 and E6 genes (Fig 1, S2 Table) and is relatively short (510-bp) in comparison to the LCR genomic region of HPV1 (979-bp). As shown in S3 Table, HPV204 and HPV1 contain two polyadenylation sites for late viral transcripts within their LCR genomic regions, whereas a single late polyadenylation site was observed in HPV63. LCR genomic regions of all *Mu*-PVs harbor a putative TATA box motif of the E6 gene promoter and two to three conserved E2-binding motifs. An 18-bp long palindrome sequence with a putative E1-binding site was identified within the LCR genomic region of each *Mu*-PV, most likely representing origin of replication [25, 48]. As described in a previous publication [26], the LCR genomic region of HPV1 contains binding sites for several transcriptional factors (e.g., NF-1, AP-1, and C/EBP). In addition, we have identified AP-1- and NF-1-binding sites in the LCR genomic region of HPV204, whereas the LCR genomic region of HPV63 contains binding sites for NF-1 and C/EBP.

### Clinical relevance and tissue tropism of HPV204, HPV1, and HPV63

To evaluate the potential clinical relevance and tissue tropism of *Mu*-PVs, we tested a representative collection of different HPV-associated clinical samples using three highly sensitive and specific quantitative type-specific RT-PCRs. Our sample collection consisted of a total of 1,006 clinical samples representing a range of the most important HPV-associated benign and malignant neoplasms (Table 2). In addition, several samples of normal mucosa and skin were tested to determine potential anatomical niches of *Mu*-PVs.

**Table 2:** The prevalence of HPV204, HPV1, and HPV63 in clinical samples.

Type of epithelia	Anatomical location/histology (sample type)	No. of samples tested	No. of HPV204-positive samples (%)	No. of HPV1-positive samples (%)	No. of HPV63-positive samples (%)
**Mucosal**	Nasopharynx (swabs)	110	0	2 (1.8)	3 (2.7)
	Oral cavity (swabs)	105	0	0	2 (1.9)
	Laryngeal papilloma (fresh tissue)	31	0	0	0
	Oral and oropharyngeal SCC (FFPE)	20	0	0	0
	Conjunctival papilloma (FFPE)	30	0	0	0
	Conjunctival carcinoma (FFPE)	34	0	0	0
	Cervix (swabs)	116	1 (0.8)	0	0
	Cervical cancer (FFPE)	59	0	0	0
**Cutaneous**	Eyebrows (hair follicles)	110	0	1 (0.9)	4 (3.6)
	Cutaneous warts (fresh tissue, FFPE)	43	1 (2.3)	21 (48.8)	9 (20.9)
	SCC (FFPE)	43	0	0	0
	BCC (FFPE)	45	0	0	0
**Mucocutaneous**	Anogenital warts (fresh tissue)	40	0	3 (7.5)	0
	Anal canal (swab)	110	4 (3.6)	0	1 (0.9)
	Penile surface (swab)	110	5 (4.5)	0	0
**Total**		1,006	11 (1.1)	27 (2.7)	19 (1.9)

SCC, squamous cell carcinoma; BCC, basal cell carcinoma; FFPE, formalin-fixed paraffin-embedded tissues.

As shown in Table 2, HPV204, HPV1, and HPV63 were detected in a total of 11/1,006 (1.1%), 27/1,006 (2.7%), and 19/1,006 (1.9%) samples, respectively, suggesting a relatively low prevalence of *Mu*-PVs in samples of various etiologies obtained from different anatomical locations. Similar to *Beta-* and *Gamma*-PVs [24, 25, 49–51], *Mu*-PVs were detected in clinical samples of cutaneous origin (eyebrow hair follicles and cutaneous warts), mucosal origin (nasopharyngeal swabs, swabs of the oral cavity, and cervical swabs negative for intraepithelial lesion or malignancy), and mucocutaneous origin (anogenital warts and swabs of the anal canal and penile surface). In accordance with a recent metagenomics study [17], our results suggest that infections with *Mu*-PVs are not strictly limited to cutaneous epithelia and that all members of the *Mu*-PV genus exhibit dual tissue tropism.

As shown in Table 2, HPV204, HPV1, and HPV63 were detected in 1/43, 21/43, and 9/43 cutaneous warts, respectively. In contrast to previous studies [52, 53], multiple HPV types were detected in 15/22 (68.2%) cutaneous warts and, interestingly, HPV1 was concurrently detected in all HPV63-positive samples (Table 3). Kohler et al. [54] have shown that, in cutaneous warts with mixed infections, clinically latent HPV infections can be discerned from a productive infection with a cutaneous wart–associated HPV type based on the viral load determination. Thus, the causative HPV types are usually present in cutaneous warts in high viral copy numbers, ranging from 51 viral copies/cell to 3.5 × 10^5^ viral copies/cell [54]. High viral copy numbers of HPV1 (range 8.5 × 10^3^ to 5.2 × 10^4^ viral copies/cell) were detected in three cutaneous warts and, because HPV1 was also the only cutaneous wart-associated HPV type identified, it most likely represented the causative agent of these warts. In addition, the viral load of HPV63 was high (3.9 × 10^2^ viral copies/cell) in one HPV63-positive cutaneous wart, suggesting that HPV63 was etiologically linked with the development of this wart, in spite of the co-presence of HPV1 in low viral copy numbers. However, the viral load of HPV1 and HPV63 was low in other cutaneous warts, ranging from 3.2 × 10^−6^ to 3.6 × 10^−2^ viral copies/cell. Similarly, the viral load of HPV204 in the only HPV204-positive cutaneous wart was extremely low (7.4 × 10^−7^ viral copies/cell), suggesting clinically latent infection with HPV204. In 11/18 (61.1%) cutaneous warts with low viral load of *Mu*-PVs, we concomitantly detected cutaneous wart–associated *Alpha*-PVs (HPV2, HPV27, and HPV57), which were the most probable etiological agents of these warts [52, 54]. As shown in Table 3, we did not detect any additional cutaneous wart-associated HPV types in seven cutaneous warts, suggesting the involvement of rarer cutaneous wart-associated HPV types, such as HPV125, HPV179, and HPV184 [24, 44] or potentially novel HPV types. Second, the low viral load of *Mu*-PVs in these lesions could be a result of the dilution of *Mu*-PV–infected cells with heterogeneous, non-targeted cell populations. Hence, fluorescent *in situ* hybridization or laser capture microdissection in combination with type-specific PCR could provide reliable determination of the causative HPV types within the lesions [55, 56]. The relatively low prevalence of HPV1- and HPV63-induced cutaneous warts in our study could be explained by the cutaneous warts’ locations, patient selection, and distinct clinical and histopathological manifestations of infection with *Mu*-PVs in different age groups [15, 16, 53]. Namely, HPV1-induced cutaneous warts are more prevalent in younger children [53], whereas all cutaneous wart samples in this study were obtained from adult patients, in whom *Alpha*-PVs are the usual etiological agents [54]. In addition, the majority of cutaneous warts were obtained from hands, whereas HPV1 and HPV63 typically cause plantar warts [16]. Moreover, to the best of our knowledge, HPV63-induced warts are rare and have been described only on the sole of the foot [10, 15, 16, 52, 53]. Interestingly, the only HPV63-positive cutaneous wart with high viral load in our study was located on a hand, suggesting HPV63 may also sporadically cause hand warts.

**Table 3:** Characteristics of HPV204-, HPV1-, and HPV63-positive tissue samples.

Clinical sample	Location	Sample type	Sample number	*Mu*-PV type^a^	Viral load (viral copies/cell)	Other HPV types
**Cutaneous warts**	Foot	Fresh tissue	ZU-7	**HPV1**	**5.2 × 10**^**4**^	/
	NA	FFPE	ZU-13	**HPV1**	**3.9 × 10**^**4**^	/
	Sole of the foot	Fresh tissue	P00057	HPV1	1.8 **×** 10^−2^	HPV57
				HPV63	2.5 **×** 10^−5^	
	Hand	Fresh tissue	P00061	**HPV1**	**8.5 × 10**^**3**^	/
	Sole of the foot	Fresh tissue	P00062	HPV1	2.1 **×** 10^−2^	HPV27
				HPV63	1.7 **×** 10^−5^	
	Hand	Fresh tissue	P00063	HPV1	5.1 **×** 10^−4^	HPV27
	Hand	Fresh tissue	P00064	HPV1	1.5 **×** 10^−4^	HPV2
				HPV63	7.3 **×** 10^−3^	
	Sole of the foot	Fresh tissue	P00065	HPV1	1.9 **×** 10^−3^	HPV2, HPV27
	Sole of the foot	Fresh tissue	P00067	HPV1	3.6 **×** 10^−2^	HPV57
				HPV63	1.1 **×** 10^−2^	
	Sole of the foot	Fresh tissue	P00070	HPV1	7.0 **×** 10^−4^	/
				HPV63	3.5 **×** 10^−6^	
	Sole of the foot	Fresh tissue	P00071	HPV1	1.9 **×** 10^−4^	/
				**HPV63**	**3.9 × 10**^**2**^	
	Hand	Fresh tissue	P00074	HPV1	8.1 **×** 10^−4^	/
				HPV63	3.7 **×** 10^−4^	
	Hand	Fresh tissue	P00076	HPV1	1.1 **×** 10^−3^	HPV57
	Hand	Fresh tissue	P00077	HPV1	6.2 **×** 10^−3^	/
				HPV63	1.4 **×** 10^−5^	
	Finger (hand)	Fresh tissue	P00078	HPV1	2.5 **×** 10^−5^	HPV57
				HPV63	3.2 **×** 10^−6^	
	Finger (hand)	Fresh tissue	P00079	HPV1	3.8 **×** 10^−4^	HPV57
	Finger (hand)	Fresh tissue	P00116	HPV204	7.4 **×** 10^−7^	HPV57
	NA	FFPE	P00162	HPV1	2.3 **×** 10^−2^	HPV57
	NA	FFPE	P00170	HPV1	6.4 **×** 10^−4^	/
	NA	FFPE	P00172	HPV1	6.7 **×** 10^−4^	/
	NA	FFPE	P00173	HPV1	4.3 × 10^−3^	/
	NA	FFPE	P00176	HPV1	4.3 × 10^−4^	/
**Anogenital warts**	NA	Fresh tissue	271	HPV1	1.9 × 10^−5^	HPV6
	NA	Fresh tissue	301	HPV1	3.2 × 10^−5^	HPV6
	NA	Fresh tissue	336	HPV1	5.9 × 10^−6^	HPV11

*Mu*-PV, *Mupapillomavirus* type; NA, not available; FFPE, formalin fixed, paraffin embedded; /, no additional HPV types detected.

^a^ The presumed *Mu*-PV types causing the cutaneous warts are written in bold.

Although it has previously been shown that eyebrow hair follicles and the oral and nasal mucosa represent important reservoirs of cutaneous HPVs [49, 50, 57, 58], the overall prevalence of *Mu*-PVs in these samples was surprisingly low in our study (Table 2), ranging from 0% to 3.6% (eyebrow hair follicles), 0% to 1.9% (swabs of the oral mucosa), and 0% to 2.7% (swabs of the nasal mucosa). In a recent study by de Koning et al. [53], a relatively high prevalence of HPV1 (59.2%) and HPV63 (25.4%) was observed in swabs of clinically normal skin obtained from children with and without cutaneous warts, suggesting the ubiquitous presence of HPV1 and HPV63. However, the use of swabs of non-hairy skin surfaces, such as palms, soles, and the forehead, might have contributed to increased detection rates observed in previous studies, because these areas contain numerous eccrine glands, which represent typical targets of HPV1 and HPV63 [15, 16, 53].

HPV1 was detected in 3/40 (7.5%) anogenital warts; however, due to low HPV1 viral copy numbers, ranging from 5.9 × 10^−6^ to 3.2 × 10^−5^ viral copies/cell (Table 3), and the co-detection of well-established causative agents of these lesions (HPV6 and HPV11), these anogenital warts could not be etiologically attributed to infection with HPV1 [54, 59]. As shown previously [60], HPV1 most probably represented a superficial contaminant, although laser capture microdissection coupled with PCR or fluorescent *in situ* hybridization should be used to determine whether HPV1 is also capable of infecting lower portions of the anogenital epithelia, such as the *stratum spinosum* and *stratum basale* [55, 56].

HPV204 was most commonly detected in swabs of the penile surface (5/110, 4.5%) and anal canal (4/110, 3.6%), suggesting its preference for anogenital mucocutaneous epithelia (Table 2). To the best of our knowledge, we detected HPV63 in the anal canal for the first time. In contrast, HPV1 was not detected in any of the swab specimens of the anal canal tested.

To the best of our knowledge, no prior studies evaluated the presence of *Mu*-PVs in cervical swab samples. Interestingly, HPV204 was the only member of the *Mu*-PV genus detected in cervical swabs negative for intraepithelial lesion or malignancy (1/116; 0.8%). Thus, HPV204 may cause clinically latent infections of the cervical mucosa as observed previously for some members of the *Gamma*-PV genus [25].

Data regarding the carcinogenic potential of HPV1 and HPV63 are scarce. In a recent study, HPV1 was detected in 1/238 BCC samples; however, no associations were found between HPV1 and an increased risk for the development of BCC [61]. In our study, none of the 201 malignant neoplasms tested positive for the presence of HPV204, HPV1, or HPV63 (Table 2). Several factors could account for these observations. First, our sample collection included a relatively large number of malignant neoplasms, which are not uniformly caused by HPV infection (e.g., SCC and BCC of the skin, conjunctival carcinoma, and oral/oropharyngeal SCC). Second, the fixation procedure in FFPE samples could have significantly degraded the DNA and, consequently, latent *Mu*-PV infection(s) with extremely low viral copy numbers might not be detected [24, 62]. However, we optimized all type-specific PCRs for *Mu*-PV detection from FFPE samples and, as shown in Table 3, viral loads were comparable regardless of the sample type (FFPE vs. fresh tissue sample). Thus, despite some limitations, our data do not support the etiologic implication of *Mu*-PVs in the development of HPV-associated malignancies.

### Genetic variability of the LCR genomic regions of HPV204, HPV1, and HPV63

In addition, we wanted to evaluate the genetic variability of *Mu*-PVs and to identify genetic variants with potential clinical or anatomical specificities. To the best of our knowledge, this is the first study to evaluate genetic variability of *Mu*-PVs by amplifying LCR genomic regions of HPV204, HPV1, and HPV63. Sanger sequencing of the obtained LCR genomic regions was performed and genetic variability of each *Mu*-PV isolate was defined as the number of SNPs/deletions per a complete LCR genomic region.

Despite using highly sensitive PCR(s), which were designed to amplify viral DNA in clinical samples with degraded DNA and low viral load, complete LCR genomic regions of HPV204 were obtained only from two swab samples of the anal canal (ENA accession numbers LT706550 and LT706551) with two HPV204 genetic variants identified. Whereas one HPV204 isolate exhibited 100% identity with the reference sequence (GenBank accession number KP769769) in its LCR genomic region, the second HPV204 isolate harbored a single SNP (A7145T) located within a putative E1-binding site (Table 4). Although the evolution of HPVs is relatively slow in comparison to some other viruses, significant variations of distinct HPV types were reported previously [2, 4, 63]. Thus, the genetic variability of HPV204 (number of SNPs/deletions per genetic variant ranging from 0/510; 0.0% to 1/510; 0.2%) was probably underestimated in our study and additional HPV204-positive samples from various anatomical locations should be evaluated to identify other potential SNPs and/or deletions.

**Table 4:** Genetic variability of the LCR genomic regions of HPV204, HPV1, and HPV63.

		Nucleotide position in the LCR																						No. of genetic variants	No. of SNPs/deletions per a complete LCR genomic region (%)
**HPV204**		7																							
		1																							
		4																							
		5																							
**Reference**	**KP769769**	**A**																							
**Anal swabs**	HPV204-LCR-1	T																						N = 1	1/510 (0.2%)
	HPV204-LCR-2	*																						N = 1	0/510 (0.0%)
**HPV1**		7	7	7	7	7	7	7	7	7	7	7	7	7	7	7	7	7	7	7	7	7	7		
		1	1	1	1	1	1	1	1	1	1	1	1	1	2	2	3	4	5	6	6	6	8		
		8	8	8	9	9	9	9	9	9	9	9	9	9	0	0	6	1	7	3	9	9	0		
		7	8	9	0	1	2	3	4	5	6	7	8	9	0	1	1	4	5	3	2	3	2		
**Reference**	**Revised V01116**	**A**	**G**	**A**	**T**	**T**	**G**	**T**	**A**	**T**	**T**	**G**	**C**	**T**	**A**	**T**	**G**	**C**	**C**	**G**	**G**	**G**	**G**		
	NC_001356^a^	*	*	*	*	*	*	*	*	*	*	*	*	*	*	*	*	*	*	*	C	C	*		
	U06714^a^	-	-	-	-	-	-	-	-	-	-	-	-	-	-	-	*	*	T	T	*	*	C		
**Nasopharyngeal swab**	HPV1-LCR-1	-	-	-	-	-	-	-	-	-	-	-	-	-	-	-	*	*	T	*	*	*	C	N = 1	17/979 (1.7%)
**Cutaneous warts**	HPV1-LCR-2	*	*	*	*	*	*	*	*	*	*	*	*	*	*	*	*	*	*	*	*	*	C	N = 1	1/979 (0.1%)
	HPV1-LCR-3	-	-	-	-	-	-	-	-	-	-	-	-	-	-	-	T	*	T	T	*	*	C	N = 1	19/979 (1.9%)
	HPV1-LCR-4	-	-	-	-	-	-	-	-	-	-	-	-	-	-	-	*	*	T	T	*	*	C	N = 13	18/979 (1.8%)
	HPV1-LCR-5	-	-	-	-	-	-	-	-	-	-	-	-	-	-	-	*	T	T	T	*	*	C	N = 2	19/979 (1.9%)
**Anogenital wart**	HPV1-LCR-5	-	-	-	-	-	-	-	-	-	-	-	-	-	-	-	*	T	T	T	*	*	C		
**HPV63**		6	6	6	6	6	6	6	6	7	7	7	0	0											
		9	9	9	9	9	9	9	9	0	1	2	0	0											
		2	3	3	3	3	3	3	3	3	2	4	6	9											
		2	1	2	3	4	5	6	7	5	4	6	4	0											
**Reference**	**X70828**	**A**	**G**	**T**	**G**	**A**	**A**	**T**	**T**	**G**	**T**	**A**	**T**	**C**											
**Cutaneous warts**	HPV63-LCR-1	T	-	-	-	-	-	-	-	A	G	C	C	G										N = 5	13/555 (2.3%)
**Eyebrow hair follicle**	HPV63-LCR-2	*	*	*	T	*	*	*	*	A	G	C	C	G										N = 1	6/555 (1.1%)

LCR, long control region; *, nucleotide matching with the PaVE reference sequence; -, nucleotide deletion at the respective nucleotide position.

^a^ Obtained from GenBank.

In a previous study, Favre et al. [18] described only one SNP (A5793C) within a partial L1 sequence of HPV1, whereas they did not evaluate genetic variability within the LCR genomic region. In our study, we obtained 18 complete LCR genomic regions of HPV1 (ENA accession numbers LT706532–LT706549) (Table 4). In comparison to HPV204, HPV1 exhibited a higher degree of genetic diversity within its LCR genomic region. In our study, we identified five HPV1 genetic variants. Within the LCR genomic region of HPV1, we identified a total of 22 nucleotide positions that harbored SNPs/deletions. The number of SNPs/deletions per genetic variant ranged from 1/979 (0.1%) to 19/979 (1.9%) and, except for a single cutaneous wart tissue sample (sample ZU-7), the LCR genomic region of all HPV1-positive samples contained a 15-bp long deletion (nucleotide positions 7,187 to 7,201), which was already described previously (GenBank accession number U06714). Interestingly, 13/18 (72.2%) sequences obtained in our study exhibited 100% sequence identity with the U06714 HPV1 sequence from GenBank, whereas four LCR sequences differed from this sequence only by a single SNP (samples 1835, ZU-13, P00061 and 271). In our sample collection of cutaneous warts, we observed a particularly prevalent genetic variant of HPV1, which was present in 13/16 (81.3%) cutaneous warts tested and contained a 15-bp long deletion together with three SNPs. The significance of this variant warrants further research, including testing of HPV1-positive samples obtained from different geographic regions. Interestingly, all SNPs/deletions identified in LCR genomic regions of HPV1 isolates were located outside viral functional domains and were not associated with specific clinical manifestations or anatomical location.

A total of six complete LCR genomic regions of HPV63 were obtained in our study (ENA accession numbers LT706552–LT706557) with two HPV63 genetic variants identified. As shown in Table 4, all HPV63 isolates obtained from cutaneous warts were identical in their LCR genomic regions and harbored six SNPs and a 7-bp long deletion (nucleotide positions 6,931–6,937). Similar to HPV1, the genetic variability of HPV63 was higher compared to HPV204 with the number of SNPs/deletions per isolate ranging from 6/555 (1.1%) to 13/555 (2.3%). None of the SNPs/deletions were located within HPV63 functional domains. Interestingly, because HPV63 isolate obtained from an eyebrow hair follicle (sample 612) differed from the reference sequence (GeneBank accession number X70828) by only six SNPs, it is suggested that HPV63 genetic variants might differ across different anatomical locations. However, our observation warrants further research on a larger collection of HPV63-positive samples.

Our evaluation of genetic variability of *Mu*-PVs was limited. Due to the low quality of samples and low viral loads of *Mu*-PVs, we could only assess their genetic variability within the LCR genomic regions. In order to provide better understanding of the prevalence and significance of SNPs and deletions, sequencing of additional genomic regions or complete HPV genomes should be performed. In addition, implementation of next-generation sequencing techniques could aid in identification of rare genetic variants of *Mu*-PVs; however, high-quality DNA is required.

In conclusion, we presented a detailed molecular characterization of a novel *Mu*-PV type HPV204 and compared it to the other members of the *Mu*-PV genus. The genome organization of all *Mu*-PVs is similar, yet each of the three members of the *Mu*-PV genus exhibit specific molecular characteristics and harbor distinct functional domains. We have systematically investigated the tissue tropism and potential clinical relevance of *Mu*-PVs by testing a large collection of HPV-associated benign and malignant neoplasms as well as samples of clinically normal mucosa and skin. *Mu*-PVs exhibit dual tissue tropism and mostly cause clinically latent infections of the skin and mucosa. In addition, HPV1 and HPV63 are etiologically implicated in the development of sporadic cases of cutaneous warts, whereas HPV204 does not appear to cause any HPV-associated neoplasms. Lastly, we have shown that the LCR genomic regions of HPV1 and HPV63 exhibit a high degree of genetic diversity; however, the clinical significance of *Mu*-PV genetic variants warrants further research.

## Supporting information

S1 Table List of PCR primers and primer pair combinations used to amplify the LCR genomic regions of HPV204, HPV1, and HPV63. (DOCX)

S2 Table Nucleotide positions of HPV204, HPV1, and HPV63 genomic regions. (DOCX)

S3 Table Characteristics of HPV204, HPV1, and HPV63 putative motifs and domains. (DOCX)
