# Supplementary material for: Molecular characterization, tissue tropism, and genetic variability of the novel Mupapillomavirus type HPV204 and phylogenetically related types HPV1 and HPV63
Source: PLoS One. 2017 Apr 20;12(4):e0175892. doi: 10.1371/journal.pone.0175892 (PMC5398564; doi:10.1371/journal.pone.0175892)
Supplement: S1 Table — (DOCX) [file pone.0175892.s001.docx]

**S1 Table. List of PCR primers and primer pair combinations used to amplify the LCR regions of HPV204, HPV1, and HPV63.**

| HPV type | Primer | Nucleotide sequence (5′–3′) | | PCR amplicon size (bp) | Nucleotide position (nt) |
| --- | --- | --- | --- | --- | --- |
| HPV204 | HPV204-LCR-F1 | | GGTTTATAGAACTGTTGATGGC | 624^a^ | 6,669–6,690 |
|  | HPV204-LCR-R1 | | AACAAATCTACAAAAGGAATGC |  | 65–44 |
|  | HPV204-LCR-F2 | | CAACTTCCACTGCCAGAACA | 618^a^ | 6,638–6,657 |
|  | HPV204-LCR-R2 | | ACTCTCTTGCACTTGTAGGTCTT |  | 28–6 |
|  | HPV204-LCR-F1 | | GGTTTATAGAACTGTTGATGGC | 271 | 6,669–6,690 |
|  | HPV204-LCR-r1 | | GCCAAAAAGGATGCTACAA |  | 6,936–6,921 |
|  | HPV204-LCR-f1 | | CTAATAAAATGCCAAGGAACA | 298 | 6,838–6,858 |
|  | HPV204-LCR-r2 | | TAACAACAATCAGTTCTTACACG |  | 7,135–7,113 |
|  | HPV204-LCR-f2 | | TTACACCTATTGTGATTCACCG | 241 | 7,052–7,073 |
|  | HPV204-LCR-R1 | | AACAAATCTACAAAAGGAATGC |  | 65–44 |
| HPV1 | HPV1-LCR-F1 | | CATGACACAACGTACTGCTACTA | 519^b^ | 6,852–6,874 |
|  | HPV1-LCR-r2 | | AGGAAAATAACACACAATCAATC |  | 7,370–7,348 |
|  | HPV1-LCR-f2 | | GCATTTTTTGTGTTCTCTGATT | 397^b^ | 7,330–7,350 |
|  | HPV1-LCR-r4 | | AAGAGTACACACCTACCGTTTTA |  | 7,726–7,704 |
|  | HPV1-LCR-f4 | | CAAACTTATCTGGTCGTGCTC | 317^b^ | 7,660–7,680 |
|  | HPV1-LCR-R1 | | TATATGGGATACAGAGGCTTTC |  | 160–140 |
|  | HPV1-LCR-F1 | | CATGACACAACGTACTGCTACTA | 289 | 6,852–6,874 |
|  | HPV1-LCR-r1 | | GCAGATGAACTATGATGTCACTAT |  | 7,140–7,117 |
|  | HPV1-LCR-f1 | | CCTTTTATTAGTGAACCATCATT | 281 | 7,090–7,112 |
|  | HPV1-LCR-r2 | | AGGAAAATAACACACAATCAATC |  | 7,370–7,348 |
|  | HPV1-LCR-f2 | | GCATTTTTTGTGTTCTCTGATT | 251 | 7,330–7,350 |
|  | HPV1-LCR-r3 | | AACAATGAAGACAATACATCCTG |  | 7,580–7,558 |
|  | HPV1-LCR-f3 | | TTGTGGCTAATCCCTTATGG | 209 | 7,518–7,536 |
|  | HPV1-LCR-r4 | | AAGAGTACACACCTACCGTTTTA |  | 7,726–7,704 |
|  | HPV1-LCR-F2 | | CCAGACTGATCCTTATAGTCAATA | 372 | 6,743–6,766 |
|  | HPV1-LCR-r1-2 | | AATGATGGTTCACTAATAAAAGG |  | 7,114–7,092 |
|  | HPV1-LCR-f1-2 | | ACATAAAATAGTCTTGGAAACCTT | 346 | 7,072–7,095 |
|  | HPV1-LCR-r2-2 | | AGGGATTAACAAAAAGAAAGGA |  | 7,397–7,418 |
|  | HPV1-LCR-f4 | | CAAACTTATCTGGTCGTGCTC | 324 | 7,660–7,680 |
|  | HPV1-LCR-R2 | | CATCAATATATGGGATACAGAGG |  | 167–145 |
| HPV63 | HPV63-LCR-F1 | | CGTAGATCCTCCAATACTACTGTG | 667^a^ | 6,847–6,870 |
|  | HPV63-LCR-R1 | | CAATAAATGGGATACGGAGAG |  | 165–145 |
|  | HPV63-LCR-F1 | | CGTAGATCCTCCAATACTACTGTG | 246 | 6,847–6,870 |
|  | HPV63-LCR-r1 | | GAGGCTATATGACTAGGCAATAAT |  | 7,092–7,069 |
|  | HPV63-LCR-f1 | | GCTGCTGTCATTTACCTGTCT | 334 | 7,041–7,061 |
|  | HPV63-LCR-r2 | | AAGAGAATCGCCTGATAGTTG |  | 26–6 |
|  | HPV63-LCR-f2 | | CAGAAACTCTTTAGGTTGCGA | 226 | 7,288–7,308 |
|  | HPV63-LCR-R1 | | CAATAAATGGGATACGGAGAG |  | 165–145 |
|  | HPV63-LCR-F2 | | CAATGCTTGAAGGTTACACAC | 260 | 6,992–7,012 |
|  | HPV63-LCR-r2-2 | | CACTTTCGTCTTTGGCAGTAA |  | 7,251–7,231 |
|  | HPV63-LCR-f2-2 | | TGGCAGATACTTCAAACAGGA | 359 | 7,116–7,137 |
|  | HPV63-LCR-R2 | | CCGAATGTACAGATGTCAGGT |  | 126–106 |

^a^ Primer pairs used to amplify the complete LCR regions of HPV204 and HPV63 in a single PCR reaction, respectively.

^b^ Three sets of overlapping PCR primer pairs used to amplify the complete LCR region of HPV1.
