# Supplementary material for: Molecular characterization, tissue tropism, and genetic variability of the novel Mupapillomavirus type HPV204 and phylogenetically related types HPV1 and HPV63
Source: PLoS One. 2017 Apr 20;12(4):e0175892. doi: 10.1371/journal.pone.0175892 (PMC5398564; doi:10.1371/journal.pone.0175892)
Supplement: S2 Table — (DOCX) [file pone.0175892.s002.docx]

**S2 Table. Nucleotide positions of HPV204, HPV1, and HPV63 genomic regions.**

|  | Nucleotide position (nt) | | |
| --- | --- | --- | --- |
| Genomic region | **HPV204** | **HPV1** | **HPV63** |
| E6 | 1–411 | 104–526 | 102–527 |
| E7 | 411–689 | 529–810 | 554–820 |
| E1 | 689–2,536 | 812–2,650 | 822–2,678 |
| E1^E4 | 689–707…3,089–3,396 | 812–827…3,157–3,561 | 822–834…3,3231–3,571 |
| E8^E2 | 1,086–1,123…3,089–3,644 | 1,200–1,231…3,200–3,797 | 1,222–1,259…3,231–3,816 |
| E2 | 2,478–3,644 | 2,592–3,797 | 2,620–3,816 |
| L2 | 3,669–5,180 | 3,898–5,421 | 3,846–5,360 |
| L1 | 5,191–6,717 | 5,432–6,940 | 5,371–6,894 |
| LCR | 6,718–7,227 | 1–103…6,941–7,816 | 1–101…6,895–7,348 |

LCR, long control region.
