# Supplementary material for: Molecular characterization, tissue tropism, and genetic variability of the novel Mupapillomavirus type HPV204 and phylogenetically related types HPV1 and HPV63
Source: PLoS One. 2017 Apr 20;12(4):e0175892. doi: 10.1371/journal.pone.0175892 (PMC5398564; doi:10.1371/journal.pone.0175892)
Supplement: S3 Table — (DOCX) [file pone.0175892.s003.docx]

**S3 Table. Characteristics of HPV204, HPV1, and HPV63 putative motifs and domains.**

| **Genomic region** | **HPV motifs and domains (consensus sequence)** | **HPV204** | **HPV1** | **HPV63** |
| --- | --- | --- | --- | --- |
| **E6** | Zinc-binding domains (CXXC(X)_29_CXXC) | aa 25–61; | aa 26–62; | aa 27–63; |
|  |  | aa 98–134 | aa 99–135 | aa 100–136 |
| **E7** | Zinc-binding domains (CXXC(X)_29_CXXC) | aa 52–87^a^ | aa 52–88 | aa 45–81 |
|  | pRB-binding site (LXCXE) | aa 24–28 | aa 24–28 | aa 22–26 |
| **E1** | Bipartite-like NLS (KRK … K(K,R)X(K,R)) | aa 76–78…113–116 | aa 74–76…109–112 | aa 79–81…113–116 |
|  | NES (LX_2-3_L(X)_2_(L,I,V)X(L,I)) | aa 97–106 | aa 94–103^a^ | aa 99–108 |
|  | Cyclin-binding motif (RXL) | aa 117–119 | aa 113–115 | aa 117–119^a^ |
|  | Cdk-phosphorylation site ((S/T)-P) | aa 82–83; | aa 80–81; | aa 85–88; |
|  |  | aa 98–99; | aa 95–96 | aa 100–101 |
|  |  | aa 107–108 |  |  |
|  | ATP-binding site (GXXXXGK(T/S)) | aa 443–450 | aa 440–447 | aa 446–453 |
| **E2** | Leucine zipper domain (L(X)_6_L(X)_6_L(X)_6_L) | aa 310–331 | aa 4–25 | absent |
| **E8** | E8 domain | aa 1–12^d^ | aa 1–12^d^ | aa 1–12^d^ |
| **L2** | Polyadenylation site (AATAAA) | nt 3,756–3,761 | nt 3,985–3,990 | nt 3,933–3,935 |
|  | Polyadenylation site (ATTAAA) | absent | nt 4,545–4,550 | absent |
|  | Furin cleavage motif (RX(K/R)R) | aa 5–8 | aa 5–8^b^ | aa 5–8 |
|  | Transmembrane-like domain (several conserved GXXXG motifs) | aa 45–67 | aa 45–67^b^ | aa 45–67 |
| **LCR** | Polyadenylation site (AATAAA) | nt 6,770–6,775; | nt 7,380–7,385; | nt 7,028–7,033 |
|  |  | nt 6,840–6,845 | nt 7,426–7,431 |  |
|  | TATA box (TATAAA) | nt 7,195–7,199 | nt 68–72 | nt 68–72 |
|  | E1-binding site (palindrome nt sequence) | nt 7,126–7,146 | nt 1–15…7,811–7,816 | nt 1–15…7,343–7,348 |
|  | E2-binding site (ACC(N)_6_GGT) | nt 7,040–7,051; | nt 7,701–7,712;^c^ | nt 7,262–7,273; |
|  |  | nt 7,070–7,081; | nt 7,775–7,786 | nt 7,308–7,319 |
|  |  | nt 7,090–7,101 |  |  |

NLS, nuclear localization signal; NES, nuclear export signal; nt, nucleotide; aa, amino acid.

^a^ aa sequences that differ from the consensus sequence.

^b^ Described by Wang et al. [27].

^c^ Described by O’Connor et al. [26].

^d^ Described by Dreer et al. [42].
